# Multidimensional Analysis of Curved Root Canal Preparation Using Continuous or Reciprocating Nickel-titanium Instruments

**DOI:** 10.2174/1874210601812010032

**Published:** 2018-01-29

**Authors:** Iussif Mamede-Neto, Álvaro Henrique Borges, Ana Helena Gonçalves Alencar, Marco Antonio Hungaro Duarte, Manoel Damião Sousa Neto, Carlos Estrela

**Affiliations:** 1 Department of Stomatologic Sciences, School of Dentistry, Federal University of Goiás, Goiânia, GO, Brazil; 2 School of Dentistry, University of Cuiabá, Cuiabá, MT, Brazil; 3 Department of Stomatologic Sciences, School of Dentistry, Federal University of Goiás, Goiânia, GO, Brazil; 4 Departament of Dentistry, Endodontics and Dental Material, Bauru Dental School, University of São Paulo, Bauru, São Paulo, Brazil; 5 Departament of Restaurative Dentistry, Dental School, University of São Paulo, Ribeirão Preto, SP, Brazil

**Keywords:** Canal transportation, Centering ability, Cone beam computed tomographic, Endodontics, Nickel-titanium instruments, Mesiobuccal root canals

## Abstract

**Objective:**

To evaluate transportation (T) and centering ability (CA) of root canal preparations using continuous or reciprocating nickel-titanium endodontic files.

**Materials and Methods:**

Ninety-six mesiobuccal root canals of mandibular first and second molars were randomly divided into 6 groups (n=16) according to the rotary file used: 1. ProTaper Next; 2. ProTaper Gold; 3. Mtwo; 4. BioRaCe; 5. WaveOne Gold; 6. Reciproc. Root canals were prepared according to manufacturer’s instructions. Cone beam computed tomography scans were obtained before and after root canal preparation. Measurements were made at six different reference points: 2, 3 and 4 mm from the apex and 2, 3 and 4 mm below furcation in different directions.

**Results:**

The greatest Mesiodistal (MD) Transportation (T) was found for Reciproc files (*p*<0.05), and the greatest buccolingual (BL) T, for Reciproc, ProTaper Gold and ProTaper Next files (*p*<0.05). The greatest Mesiodistal (MD) Centering Ability (CA) was found for BioRaCe files (*p*<0.05), and the greatest Buccolingual (BL) CA, for BioRaCe and Mtwo files (*p*<0.05).

**Conclusion:**

All systems produced root canal transportation. No file system achieved perfect CA of root preparation. Reciproc files had the greatest MD T and BL T. BioRaCe files had the greatest MD CA, whereas BL CA was similar for BioRaCe and Mtwo files.

## 1 INTRODUCTION

The purpose of root canal preparation is to clean and shape the pulp cavity while preserving its original shape and curvature and the position of the apical foramen [1-3]. Procedural iatrogenic errors in the preparation of curved root canals, such as zips, perforations, decentralized root canals, apical foramen transport, are risk factors that may lead to root canal treatment failure [4, 5].

The original shape of curved root canals is better preserved when they are prepared with flexible nickel-titanium files instead of stainless steel files [6]. The centering ability of continuous and reciprocating nickel-titanium files is better than that of stainless steel files and, consequently, their root canal transportation is reduced [7-9].

The cross-section, rake angle, taper, number of flutes and radial land of nickel-titanium files have specific characteristics [10]. The mechanical properties and the behavior of nickel-titanium alloys may vary according to their chemical composition and heat treatment during manufacture [11].

The effectiveness of some of these files has been analyzed considering geometry preservation and root canal transportation, endodontic file fracture and dentin fracture [3, 12-18]. Recent studies have demonstrated their satisfactory results in curved root canal enlargement, transportation and centering [7, 10, 12]. The imaging studies most often used to determine procedural operative errors are periapical radiographs [4, 14, 19], scanning electronic microscopy [20], micro-computed tomography [16, 21, 22], and cone-beam computed tomography (CBCT) [1, 4, 7, 12, 23, 24].

The parameters to determine morphological changes in drilled areas after root canal preparation should be carefully analyzed. Transportation and centering ability during preparation at the cervical and apical levels in the mesiodistal and buccolingual directions on CBCT images may be incorporated into a method with a solid referential basis. Thus, this study evaluated the transportation and centering ability of root canal preparation using continuous or reciprocating nickel-titanium instruments with multidimensional imaging method.

## 2 MATERIALS AND METHODS

### 2.1 Sample Selection and Preparation

This study included human permanent mandibular first and second molars obtained from the Dental Urgency Department of the School of Dentistry of the Federal University of Goiás. The specimens were stored in a solution of 0.2% thymol. This study was approved by the Ethics in Research Committee of the Federal University of Goiás, Goiânia, Brazil (CAAE: 53712816.1.0000.5083) and it has been conducted in full accordance with the World Medical Association Declaration of Helsinki.

Preoperative periapical radiographs of each tooth were used for sample selection according to selection criteria. A platform was used to standardize tooth images. A Spectro X70 Electronic x-ray unit (Dabi Atlante, Ribeirão Preto, Brazil) and a RVG 5100 digital sensor (Carestream Dental, Atlanta, GA) were used for lateral radiographs. All images were evaluated using the RVG 5100 software (Carestream Dental, Atlanta, GA).

Baseline images were acquired using a Prexion 3D scanner (PreXion 3D Inc., San Mateo, CA). Image thickness was 0.110 mm (size: 1.170 mm X 1.570 mm X 1.925 mm), 81.00 mm X 75 mm FOV, 0.100 mm voxel, 33.5 s scan time (1,024 matrix), 90 KVP and 4 mA. The images were analyzed using the CT scanner software (Prexion 3D Viewer, TeraRecon Inc, Foster City, CA) on an Intel i7 2.86 GHz (Intel Corp., Santa Clara, CA) Windows 8 Professional (Microsoft Corp., Redmond, WA) workstation equipped with an NVIDIA GeForce 6200 turbo cache videocard (NVIDIA Corp., Santa Clara, CA) and a 1600 X 1200 pixels ELZO-Flexscan S2000 monitor (ELZO NANAO Corp., Hakusan, Japan).

Inclusion criteria were: teeth with no internal or external root resorptions, fractures or calcifications; and with an intact pulp cavity and fully formed roots. Teeth were excluded if their length was greater than 22 mm, or if mesiobuccal canals had more than one apical foramen or a curvature radius smaller than 4 mm and greater than 9 mm, according to the method described by Estrela *et al*. [25].

To determine the curvature radius of curved root canals, two 6 mm straight lines were superimposed to the root canal image: the primary line defined the apical region, and the secondary, the middle and cervical thirds. Regardless of the total length of the secondary line, only the 6 mm closest to the primary line were used for the measurements. The midpoint of each line was determined, and two perpendicular lines were drawn to a central point of a circumference, the circumcenter. The distance from circumcenter to the midpoint of each line (primary and secondary) was the circle radius, which represented the magnitude of the curvature [25].

### 2.2 Root Canal Preparation

The teeth were rinsed under running water to fully remove thymol solution and then dried with absorbing paper towels. After that, they were immersed in 5% sodium hypochlorite for 30 min to remove all organic tissues.

The study sample comprising 96 mesiobuccal canals of mandibular molars was randomly distributed into 6 groups (n=16) of different rotary systems: 1. ProTaper Next X4 (Dentsply/Maillefer, Switzerland); 2. Protaper Gold F4 (Dentsply/Maillefer, Switzerland); 3. Mtwo 40/.04 (VDW Dental, Germany); 4. BioRaCe BR5 (FKG Dentaire, Switzerland); 5. WaveOne Gold Large (Dentsply/Maillefer, Switzerland); and 6. Reciproc R40 (VDW Dental, Germany).

A high-speed handpiece, round diamond burs (#1013, #1014; KG Sorensen, Barueri, Brazil) and an Endo Z bur (Dentsply/Maillefer, Switzerland) were used for coronal flaring under irrigation. After that, the mesiobuccal root canals were explored and their contents were removed using K-file #10 and K-file #15 stainless steel handfiles (Dentsply/Maillefer, Switzerland). The cervical third was prepared using the files for this area in each system under study. Working length was determined using a K-file #15 and confirmed by visualization of the file tip through the apical foramen. The file was pulled back one millimeter to determine actual working length.

### 2.3 Root Canals were Prepared According to Manufacturer’s Instructions

In reciprocating rotary files, it were used a single-file for each root canal preparation. For the others groups (continuous rotary files), it was used a sequence of instruments until to the diameter corresponding to ProTaper Next X4, Protaper Gold F4, Mtwo 40/.04, BioRaCe BR5 for each root canal preparation. An X-Smart Plus engine (Dentsply/Maillefer, Switzerland) was used for all files, and the root canals were irrigated with 2.5% sodium hypochlorite freshly prepared before use (Fitofarma, Goiânia, Brazil) and delivered using a Navitip irrigation tip (Ultradent Products Inc., South Jordan, UT). During canal cleaning, 30 mL of irrigant was used. Canal preparation was completed when the last file reached working length in free rotation and then removed. Patency was checked with a K #15 file. After instrumentation was completed, the root canals were dried with absorbing paper points of the same caliber as the last file and then irrigated with 5 mL of 17% EDTA for 3 min. After the last irrigation with 5 mL of 2.5% NaOCl, the root canals were dried again.

Each endodontic file was used to prepare only one root canal. All the root canals were prepared by an endodontist with over 15 years’ experience.

After preparation, final CBCT scans were obtained to evaluate transportation and centering ability of the endodontic files. The same protocol described for the acquisition of baseline images was followed, and the image synchronization tool of the Prexion software (Prexion 3D Viewer, TeraRecon Inc, Foster City, CA) was used for the axial, coronal and sagittal views.

The following reference points were used for measurements on the root canal images: 1- 2 mm short of the apex; 2- 3 mm short of the apex; 3- 4 mm short of the apex; 4-2 mm below furcation; 5-3 mm below furcation; 6-4 mm below furcation. Navigation on the axial view of synchronized images started at the root apex, both on baseline and final images, and moved to the measurement points on the apical third. For the measurements on the cervical third, navigation started at furcation and moved down up to 4 mm. To facilitate measurements, the enlargement, brightness and contrast tools available in the software were used.

### 2.4 Analysis of Root Canal Transportation

**Fig. (1):**
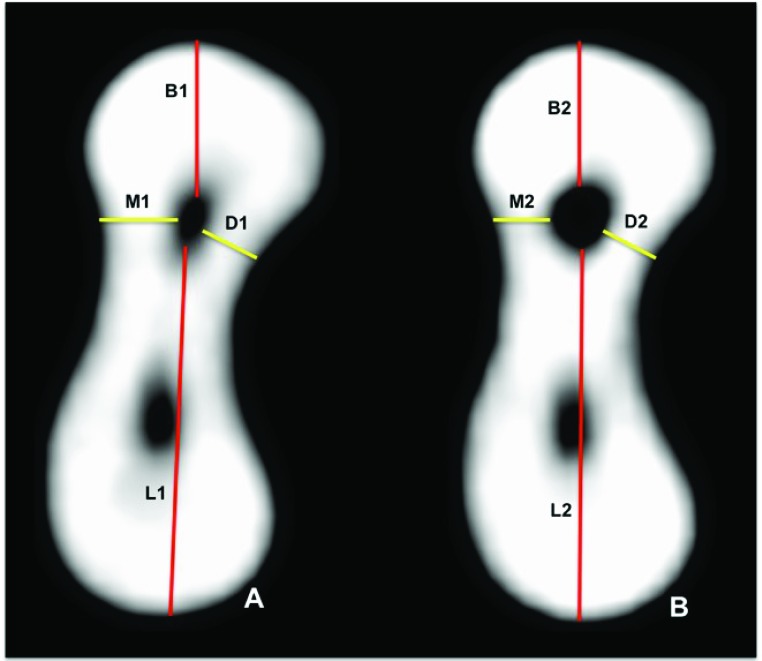
Cone-bean computed tomography scan (axial view) of mesial root of mandibular molar at 3 mm short of apex, point for measurement of mesiodistal and buccolingual distances to determine root canal transportation: (A) before root canal preparation; (B) after root canal preparation.

The evaluation of images to determine root canal transportation (T) followed the method described by Gambill *et al*. [1]. Root canal transportation, determined in the mesiodistal and buccolingual directions at the six points described above, corresponded to the variation, in millimeters, of the deviation from the central axis of the root canal after preparation. Mesiodistal transportation was the shortest distance between the mesial and distal walls of the root canal and the external mesial and distal surface before (M1 and D1) and after (M2 and D2) root canal preparation (Fig. **1**).

In the same way, buccolingual root canal transportation was the shortest distance between the images of the buccal and lingual wall of the root canal and the external buccal and lingual surfaces before (B1 and L1) and after (B2 and L2) root canal preparation (Fig. **1**). The images were analyzed using the CT scanner software (Prexion 3D Viewer, TeraRecon Inc, Foster City, CA) on an Intel i7 2.86 GHz (Intel Corp., Santa Clara, CA) Windows 8 Professional (Microsoft Corp., Redmond, WA) workstation equipped with an NVIDIA GeForce 6200 turbo cache videocard (NVIDIA Corp., Santa Clara, CA) and a 1600 X 1200 pixels ELZO-Flexscan S2000 monitor (ELZO NANAO Corp., Hakusan, Japan). Measurements were made by one examiner at two time points, and agreement was greater than 80% according to kappa statistics (K=0.882).

Mesiodistal (MD) and buccolingual (BL) transportation was calculated using the formula below:

T(MD) = (M1 – M2) – (D1 – D2) and T(BL) = (B1 – B2) – (L1 – L2). A negative T(MD) result indicated distal transportation, whereas a positive number indicated mesial transportation; a result of zero indicated no transportation. A negative T(BL) result indicated lingual transportation, whereas a positive number indicated buccal transportation; a result of zero indicated no transportation.

### 2.5 Analysis of Centering Ability of Root Canal Preparation

Centering ability (CA) was analyzed using the method described by Gambill *et al*. [1], who defined centering ability as the capacity of an endodontic file of preserving the central axis of the root canal. CA was calculated using the distances measured to define transportation.

Mesiodistal and buccolingual CA were calculated using the following formula:

CA(MD) = (M1-M2) / (D1-D2) or CA(MD) = (D1-D2) / (M1-M2)

CA(BL) = (B1-B2) / (L1-L2) or CA(BL) = (L1-L2) / (B1-B2)

The numerator was the smallest difference between distances before and after preparation. When the result was one (CA = 1), mesiodistal or buccolingual CA was perfect, and the closest it was to zero (0 ≥ CC ≤ 0.999), the poorest the centering ability was.

### 2.6 Statistical Analysis

The original transportation and CA values were entered in a Microsoft Office Excel spreadsheet (Microsoft Corporation, Redmond, WA) and later exported to the IBM SPSS 20.0 software (SPSS Inc., Nova York, NY) for statistical analyses. Data were described as median, minimum and maximum values, and compared between files using the Kruskal-Wallis test, and compared between specific points in the root canal using the Friedman test. The Bonferroni correction was used to adjust comparisons. The level of significance was set at 5%.

## 3 RESULTS

**Fig. (2):**
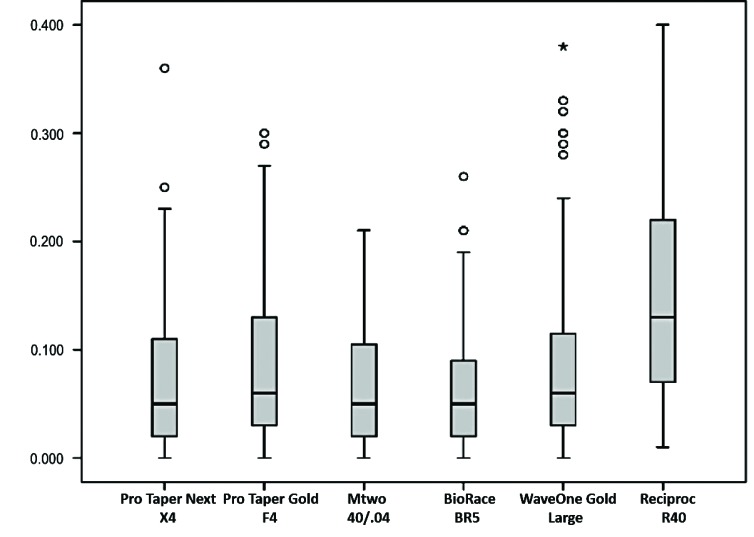
Mesiodistal root canal transportation (mm).

All files under study had positive median T(MD), that is, all produced mesial transportation (Fig. **2**). The lowest T(MD) was found for Mtwo, ProTaper Next, BioRaCe, ProTaper Gold and WaveOne Gold systems, and there were no significant differences between them. The greatest T(MD) was found for Reciproc, and the result was significantly different from those found for the other systems (*p*<0.05) (Table **1**). However, when the six points were evaluated, there were no significant differences between systems at 2 mm short of the apex.

**Fig. (3):**
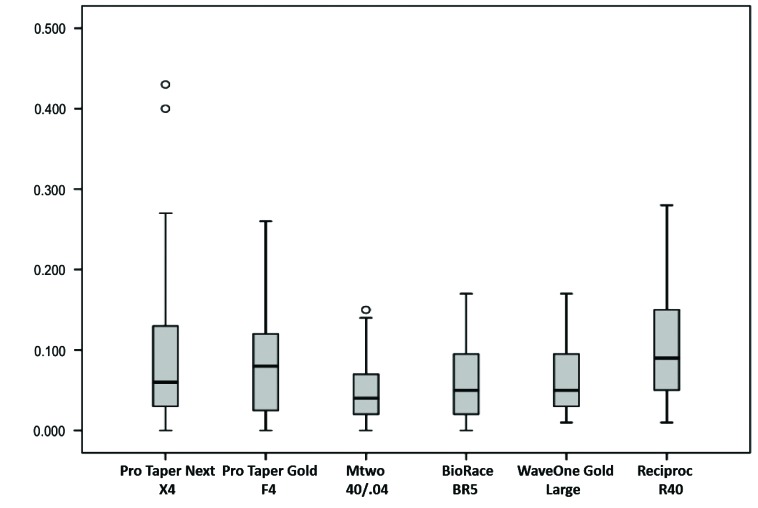
Buccolingual root canal transportation (mm).

The analysis of root canal T(BL) revealed that the median values of all systems under study were positive, that is, transportation occurred in the buccal direction (Fig. **3**). The lowest T(BL) values were found for the Mtwo, BioRaCe and WaveOne Gold systems, with no significant differences between them; the highest T(BL) values were found for the Reciproc, ProTaper Gold and ProTaper Next systems, and their differences from the other systems were statistically significant (*p*<0.05) (Table **1**). Although there were no significant differences between files at 2 mm and 3 mm short of the apex and at 2 mm below furcation, the Reciproc system had a greater T(BL) than the other systems at 4 mm short of the apex and at 3 mm below furcation (*p*<0.05). However, the Reciproc and ProTaper Gold systems had a greater T(BL) than the other systems at 4 mm below furcation, and the difference was significant (*p*<0.05).

**Fig. (4):**
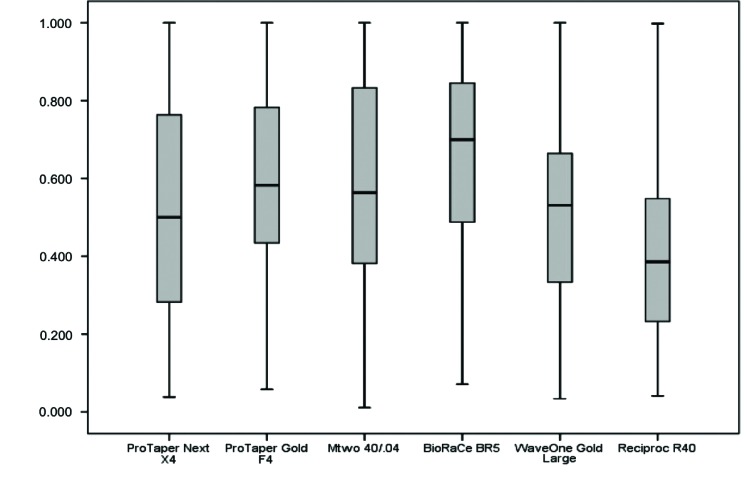
Mesiodistal centering ability of root canal preparation.

No system under study had median values of CA(MD) equal to one, that is, there was no perfect centering ability (Fig. **4**). The lowest CA(MD) value was found for the Reciproc system, and the difference from the other systems was statistically significant (*p*<0.05), whereas the BioRaCe system had the highest values, and the difference between systems was also statistically significant (*p*<0.05) (Table **2**). However, when findings for the six measurement points were compared, no significant differences were found between systems at 2 mm and 3 mm short of the apex ad at 2 mm below furcation. The highest CA(MD) value at 4 mm short of the apex was found for the BioRaCe system, and the values were statistically different from those found for the ProTaper Next and Reciproc systems (*p*<0.05). The lowest CA(MD) at 3 mm below furcation was found for the Reciproc system, and the difference from the other systems was significant (*p*<0.05). The WaveOne Gold and Reciproc systems had the lowest CA(MD) values at 4 mm below furcation, and the differences from the other systems were statistically significant (*p*<0.05).

**Fig. (5):**
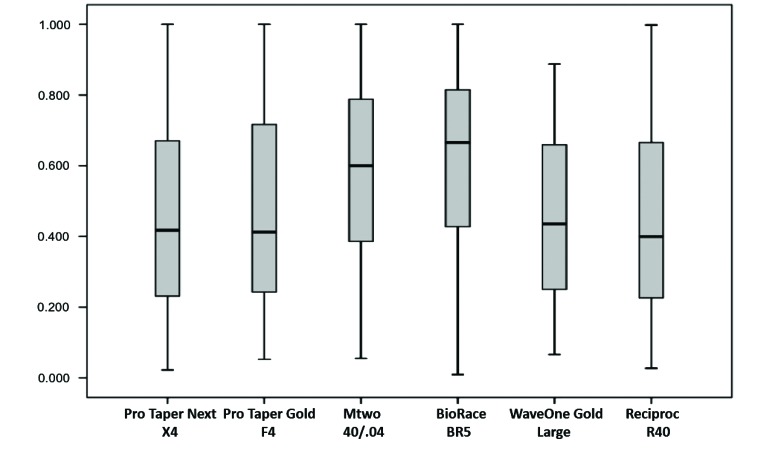
Buccolingual centering ability of root canal preparation.

The analysis of CA(BL) revealed that no system under study had median values equal to one, that is, no system had perfect centering ability (Fig. **5**). The highest CA(BL) values were found for the BioRaCe and Mtwo systems, and the differences from the other systems were statistically significant (*p*<0.05) (Table **2**). The analysis of CA(BL) values at 2 mm, 3 mm and 4 mm short of the apex and at 2 mm and 3 mm below furcation did not reveal any statistically significant differences between systems. However, the ProTaper Next system had the lowest CA(BL) value at 4 mm below furcation, and this result was statistically different from the value found for Mtwo (*p*<0.05).

The measurements at the six points before and after root canal preparation revealed the lowest T(MD) was found at 2 mm short of the apex, and this finding was statistically significant (*p*<0.05) (Table **3**); and the lowest T(BL) was found at 2 mm and 3 mm short of the apex, but the differences were not statistically significant (Table **4**).

The highest CA(MD) values were found at 4 mm short of the apex, and the highest CA(BL), at 3 mm below furcation, but the differences between systems were not statistically significant (Tables **5** and **6**).

Figs. (**6** and **7**) show CBCT axial views of the cervical and apical thirds of the mesiobuccal canals before and after preparation according to the type of NiTi file used.

## 4 DISCUSSION

Changes in root canal shape in the cervical and apical thirds were found for all the files under study. Transportation and CA were determined using a model previously described by Gambill *et al*. [1], who used a model of mesiodistal measurements of axial plans from the apex of single-rooted teeth whose curvature was less than 10 degrees. Several studies have adopted this method to evaluate transportation and CA after the preparation of curved root canals [7, 12, 24].

The present study used mesiobuccal root canals of mandibular molars with a curvature radius greater than 4 mm and shorter than 8 mm [25]. Two areas of clinical reference, 2 mm to 4 mm below furcation and 2 mm to 4 mm short of the apex, and two directions, mesiodistal and buccolingual, were analyzed using high resolution CBCT images. Our study used the image synchronization program (Prexion software) allowing a correct analysis of the root canal preparation. This fact allowed to verify that some areas of risk of excessive wear. Several studies used different methods to evaluate the preservation of the shape of curved root canals after preparation with stainless steel files or continuous or reciprocating NiTi files: artificial canals [26], scanning electronic microscopy [20], periapical radiographs of human teeth [10, 14, 19, 27], micro-computed tomography [8, 13, 15, 16, 21, 22, 28-30], and CBCT [1, 7, 12, 23, 24].

CBCT, a non-destructive procedure, may be a potential method for an accurate evaluation of root canal geometry because of the different plans used for three-dimensional analysis and the axial views, which avoid the superimposition of structures [1, 23, 25].

This study found that there were both mesiodistal and buccolingual changes in the shape of the root canal (Figs. **2** and **3**). The morphological buccolingual changes after the preparation of curved root canals may be accurately evaluated using CBCT images. The major morphological changes were mesiodistal.

The results found for the ProTaper Next, ProTaper Gold, WaveOne Gold, Mtwo, BioRaCe and Reciproc files in the analysis of transportation and centering ability after the preparation of curved root canals are in agreement with previous studies, which concluded that they preserved the shape of root canals [13, 15, 19, 27]. Several studies found that the results of shape preservation after root canal preparation were often favorable for new endodontic files, but their methods, measurement points along the root thirds and the amount of enlargement that they used were different from each other [10, 20, 22]. In this study, root canals were prepared using of the following diameters and tapers: 40/.04, 40/.06 and 45/.05. The discussion about how much a root canal should be enlarged remains to be investigated in future studies. WU *et al*. [31] found that canal preparation depends on the morphology and thickness of the root canal walls, as well as on the taper of the file selected. At the same time, other studies reported that not all the walls are touched by the files during root canal preparation [3, 23, 31-33]. In this study, the apex was enlarged using files whose diameter was larger than 350 μm, and, therefore, it was possible to compare the performance of files whose diameter and taper were larger in curved root canals. Moreover, these files act on a larger area of the root canal walls, which facilitates the penetration of the irrigation tip and, consequently, the action of antimicrobials. Despite the amount of enlargement, the files used for root canal preparation had satisfactory transportation and CA results (Fig. **2**-**5**). An earlier study [34] found that there were no significant differences in transportation and CA after 35/.02 and 50/.02 RaCe files were used for root canal preparation.

The files under evaluation in this study were selected according to their morphological characteristics, mechanical properties, chemical composition and capacity of preserving the original shape of curved root canals because of their flexibility [3, 7-11]. The endodontic files included in this study have different cross-sections, diameters, tapers, types of alloy and tip designs [8, 10, 13, 14, 19]. The comparison of the ProTaper files (ProTaper Universal, ProTaper Next and ProTaper Gold) to each other revealed that the ProTaper Gold files, because of the technology used for their manufacture and thermal treatment, had better cyclic fatigue and flexibility and produced less apical transportation than the ProTaper Universal and ProTaper Next files [13]. Mtwo files are manufactured using the conventional nickel-titanium alloy. BioRaCe files, when used to prepare curved root canals, do not change the original root canal anatomy because of their triangular cross section, associated with their flexibility, and their alternating cutting edges, which avoid self-threading [14, 34]. The angle of the taper of Reciproc and WaveOne files is high in their apical 3 mm (D0 - D3) [10, 14, 18]. In addition to their cross-sectional design, another important characteristic is a result of the use of the m-wire alloy, responsible for their greater flexibility [10, 12, 17].

Method variations between studies, particularly the amount of apical enlargement, evaluation criteria and evaluation tools justify the differences found. However, there seems to be a consensus about the fact that the use of NiTi rotary files results in low apical transportation and good centering during root canal preparation.

Bürklein *et al*. [19] evaluated the shaping effectiveness of rotary flies with a diameter of 400 μm (ProTaper Universal, ProTaper Next, BT-RaCe and Mtwo) used to prepare severely curved root canals. The files were safe and preserved root canal curvature. Garcia *et al*. [27] used periapical radiographs to evaluate transportation of ProFile and RaCe files in the preparation of mandibular molar canals. The size (diameter and taper) of the files used for apical enlargement was 40/.04. They did not find any significant differences in apical transportation. Yang *et al*. [15] compared the geometry of root canals prepared with ProTaper Universal and Mtwo files. Both files preserved canal geometry during preparation. Gagliardi *et al*. [13] used micro-computed tomography imaging to evaluate transportation and CA in curved root canals of mandibular molars prepared with ProTaper Gold, ProTaper Next and ProTaper Universal files. Apical enlargement corresponded to 250 μm files. ProTaper Gold and ProTaper Next files produced less transportation and had better CA than the ProTaper Universal files.

Reciprocating rotary files also had satisfactory transportation and CA results in the preparation of curved root canals at the different points evaluated (cervical and apical thirds). Capar *et al.* [12] used CBCT to compare the effects of OneShape, ProTaper Universal, ProTaper Next, Reciproc R25, Twisted File Adaptive and WaveOne Primary files on transportation and CA when used to prepare curved canals. All files had similar transportation and CA. Carvalho *et al*. [7] evaluated apical transportation and CA of the reciprocating system Reciproc associated with different glide path techniques. Root canals prepared using a glide path technique had minimal apical transportation, and the Reciproc system had good CA in the preparation of root canals. Saber *et al*. [14] compared the shaping ability of the WaveOne^®^ Primary, Reciproc R25 and OneShape systems in the preparation of severely curved root canals of extracted human molars. All systems were safe. WaveOne Primary and Reciproc R25 files were more efficient in preserving the original canal curvature. Bürklein *et al*. [10] used periapical radiographs to compare Mtwo 30/.05, ProTaper Universal F3, Reciproc R25 and WaveOne Primary files used to shape curved root canals of extracted teeth. All systems preserved the original root canal curvature and were safe to use. Amaral *et al*. [22] used micro-computed tomography to evaluate transportation and CA in root canals prepared with WaveOne Primary files alone or together with previous apical and cervical enlargement. The association with previous cervical or apical enlargement resulted in a reduction of transportation and in better CA than the use of WaveOne files alone.

This present study found that the preparation of curved root canals using continuous (ProTaper Next, ProTaper Gold, Mtwo and BioRaCe) or reciprocating NiTi files (WaveOne Gold and Reciproc) had low apical transportation and satisfactory CA in the preparation of curved root canals. The maintenance of the apical limit during instrumentation in rotary and reciprocating modes was also evaluated previously [35]. Seventy-two human uniradicular mandibular premolars were prepared with # F4 ProTaper and # R40 Reciproc instruments. The results show that the devices were able to control the apical limit of the instrumentation independent of the kinematics and working length applied. Another relevant finding of our study was the degree of mesiodistal and buccolingual enlargement of curved root canals with a curvature radius greater than 4 mm and shorter than 8 mm, which was indicative of satisfactory shaping, both in the cervical and apical thirds.

Contemporary endodontics has developed good standards for shaping with the new NiTi rotary systems, which has directly affected the quality of obturations and, therefore, the success and survival of root canal treatments. However, the challenge and enigma of biofilm destruction in areas that the files and the antimicrobials do not reach remain as problems to be further investigated.

In summary, all the continuous and reciprocating files produced root canal transportation, and no file had perfect CA. The greatest mesiodistal transportation was found for the Reciproc system, and buccolingual transportation, for the Reciproc, ProTaper Gold and ProTaper Next systems. The BioRaCe system had the best mesiodistal CA, and the BioRaCe and Mtwo had similar buccolingual CA. The lowest mesiodistal transportation was found at 2 mm short of the apex. The best mesiodistal CA was found at 4 mm short of the apex, and the best buccolingual CA, at 3 mm below furcation.

## CONCLUSION

All systems produced root canal transportation. No file system achieved perfect CA of root preparation. Reciproc files had the greatest MD T and BL T. BioRaCe files had the greatest MD CA, whereas BL CA was similar for BioRaCe and Mtwo files.

## Figures and Tables

**Fig. (6):**
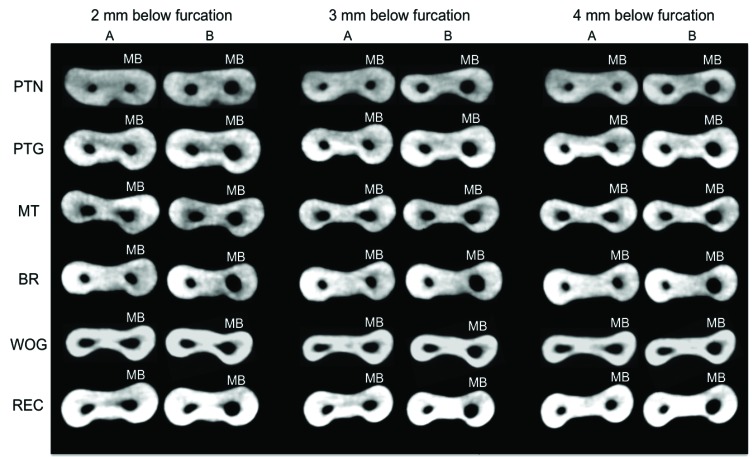
Cone-beam computed tomography scan (axial view) of cervical third of mesiobuccal root canals. A – before preparation. B – after preparation. PTN - ProTaper Next^®^; PTG - ProTaper Gold^®^; MT - Mtwo^®^; BR - BioRaCe^®^; WOG - WaveOne Gold^®^; REC - Reciproc^®^; MB – mesiobuccal canal.

**Fig. (7):**
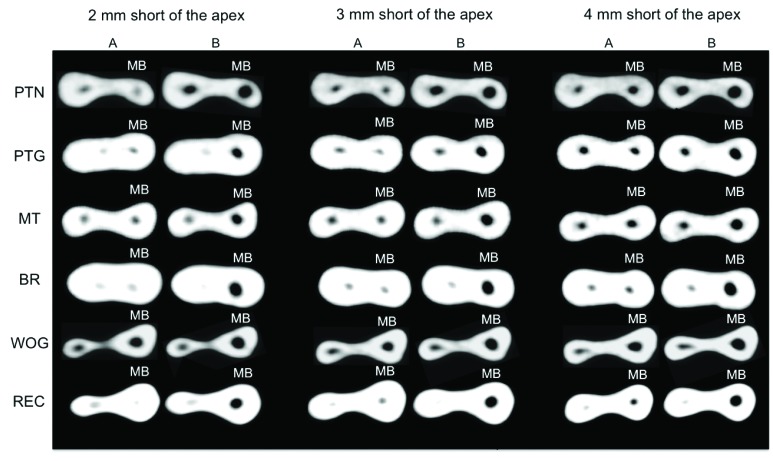
Cone-beam computed tomography scan (axial view) of apical third of mesiobuccal root canals. A – before preparation. B – after preparation. PTN -ProTaper Next^®^; PTG - ProTaper Gold^®^; MT - Mtwo^®^; BR - BioRaCe^®^; WOG - WaveOne Gold^®^; REC - Reciproc^®^; MB – mesiobuccal canal.

**Table 1:** Median, minimum and maximum values of T(MD) and T(BL) (mm).

**Files**	**–**	**T(MD)**	**T(BL)**
–	Median	0.050^a^	0.060^b, c^
ProTaper Next	Minimum	0.000	0.000
–	Maximum	0.360	0.430
–	Median	0.060^a^	0.080^b. c^
ProTaper Gold	Minimum	0.000	0.000
–	Maximum	0.300	0.260
–	Median	0.050^a^	0.040^a^
Mtwo	Minimum	0.000	0.000
–	Maximum	0.210	0.150
–	Median	0.050^a^	0.050^a b^
BioRaCe	Minimum	0.000	0.000
–	Maximum	0.260	0.170
–	Median	0.060^a^	0.050^a.b^
WaveOne Gold	Minimum	0.000	0.010
–	Maximum	0.380	0.170
–	Median	0.130^b^	0.090^c^
Reciproc	Minimum	0.010	0.010
–	Maximum	0.400	0.280

*In each column, medians with a common superscript letter are not statistically different (*p*<0.05).

**Table 2:** Median, minimum and maximum values of mesiodistal centering ability [CA(MD)] and buccolingual centering ability [CA(BL)].

**Files**	**–**	**CA(MD)**	**CA(BL)**
–	Median	0.500^b^	0.418^b^
ProTaper Next	Minimum	0.038	0.022
–	Maximum	1.000	1.000
–	Median	0.583^b^	0.413^b^
ProTaper Gold	Minimum	0.000	0.052
–	Maximum	0.300	1.000
–	Median	0.563^b^	0.600^a^
Mtwo	Minimum	0.011	0.055
–	Maximum	1.000	1.000
–	Median	0.700^a^	0.666^a^
BioRaCe	Minimum	0.071	0.009
–	Maximum	1.000	1.000
–	Median	0.531^b^	0.436^b^
WaveOne Gold	Minimum	0.034	0.066
–	Maximum	1.000	0.888
–	Median	0.386^c^	0.400^b^
Reciproc	Minimum	0.041	0.027
–	Maximum	0.998	0.998

*In each column, medians with a common superscript letter are not statistically different (*p*<0.05).

**Table 3:** Median, minimum and maximum values of T(MD) at different points in root canal.

**–**	2 mm Short of Apex	3 mm Short of Apex	4 mm Short of Apex	2 mm Below Furcation	3 mm Below Furcation	4 mm Below Furcation
Median	0.030^a^	0.055^b^	0.055^b^	0.075^b^	0.100^c^	0.130^c^
Minimum	0.000	0.000	0.000	0.000	0.000	0.000
Maximum	0.140	0.190	0.290	0.270	0.400	0.390

*In each column, medians with a common superscript letter are not statistically different (*p*<0.05).

**Table 4:** Median, minimum and maximum values of T(BL) at different points in root canal (mm).

**–**	2 mm Short of Apex	3 mm Short of Apex	4 mm Short of Apex	2 mm Below Furcation	3 mm Below Furcation	4 mm Below Furcation
Median	0.050^a^	0.050^a^	0.070^a^	0.070^a^	0.060^a^	0.060^a^
Minimum	0.000	0.000	0.000	0.000	0.000	0.000
Maximum	0.220	0.220	0.430	0.210	0.270	0.270

*In each column, medians with a common superscript letter are not statistically different (*p*<0.05).

**Table 5:** Median, minimum and maximum values of CA(MD) at different points in root canal.

**–**	2 mm Short of Apex	3 mm Short of Apex	4 mm Short of Apex	2 mm Below Furcation	3 mm Below Furcation	4 mm Below Furcation
Median	0.554^a^	0.500^a^	0.571^a^	0.548^a^	0.500^a^	0.448^a^
Minimum	0.011	0.041	0.045	0.034	0.052	0.048
Maximum	1.000	1.000	1.000	1.000	1.000	1.000

*In each column, medians with a common superscript letter are not statistically different (*p*<0.05).

**Table 6:** Median, minimum and maximum values of CA(BL) at different points in root canal.

**–**	2 mm Short of Apex	3 mm Short of Apex	4 mm Short of Apex	2 mm Below Furcation	3 mm Below Furcation	4 mm Below Furcation
Median	0.500^a^	0.500^a^	0.500^a^	0.500^a^	0.570^a^	0.547^a^
Minimum	0.027	0.026	0.022	0.025	0.009	0.040
Maximum	1.000	1.000	1.000	1.000	1.000	1.000

*In each column, medians with a common superscript letter are not statistically different (*p*<0.05).
